# Synthesis of 2,6-Diamino-Substituted Purine Derivatives and Evaluation of Cell Cycle Arrest in Breast and Colorectal Cancer Cells

**DOI:** 10.3390/molecules23081996

**Published:** 2018-08-10

**Authors:** Bartolomeo Bosco, Andrea Defant, Andrea Messina, Tania Incitti, Denise Sighel, Angela Bozza, Yari Ciribilli, Alberto Inga, Simona Casarosa, Ines Mancini

**Affiliations:** 1 Centre for Integrative Biology (CIBIO), University of Trento, Via Sommarive 9, 38123 Trento, Italy; bartolomeo.bosco@unitn.it (B.B.); andrea.messina@unitn.it (A.M.); tania.incitti@gmail.com (T.I.); denise.sighel@unitn.it (D.S.); angela.bozza@aulss8.veneto.it (A.B.); yari.ciribilli@unitn.it (Y.C.); alberto.inga@unitn.it (A.I.); 2 Laboratory of Bioorganic Chemistry, Department of Physics, University of Trento, Via Sommarive 14, 38123 Trento, Italy; andrea.defant@yahoo.it

**Keywords:** reversine, microwave-assisted synthesis, molecular docking, cell cycle arrest, endoreduplication, p53

## Abstract

Reversine is a potent antitumor 2,6-diamino-substituted purine acting as an Aurora kinases inhibitor and interfering with cancer cell cycle progression. In this study we describe three reversine-related molecules, designed by docking calculation, that present structural modifications in the diamino units at positions 2 and 6. We investigated the conformations of the most stable prototropic tautomers of one of these molecules, the *N*6-cyclohexyl-*N*6-methyl-*N*2-phenyl-7*H*-purine-2,6-diamine (**3**), by Density Functional Theory (DFT) calculation in the gas phase, water and chloroform, the last solvent considered to give insights into the detection of broad signals in NMR analysis. In all cases the H*N*(9) tautomer resulted more stable than the H*N*(7) form, but the most stable conformations changed in different solvents. Molecules **1**–**3** were evaluated on MCF-7 breast and HCT116 colorectal cancer cell lines showing that, while being less cytotoxic than reversine, they still caused cell cycle arrest in G2/M phase and polyploidy. Unlike reversine, which produced a pronounced cell cycle arrest in G2/M phase in all the cell lines used, similar concentrations of **1**–**3** were effective only in cells where p53 was deleted or down-regulated. Therefore, our findings support a potential selective role of these structurally simplified, reversine-related molecules in p53-defective cancer cells.

## 1. Introduction

Pluripotent cells play a relevant role in regenerative medicine, due to their capability of differentiating into many cell types. Pluripotency can be achieved in vitro, by over-expressing specific genes or by chemical induction, using a combination of synthetic molecules [1]. Among them the 2,6-diamino-substituted purine reversine (*N*6-cyclohexyl-*N*2-(4-morpholinophenyl)-7*H*-purine-2,6-diamine), firstly synthesized by Schultz and coworkers [2], is a promising molecule. In fact, it induces de-differentiation of C2C12 murine myoblasts and allows their subsequent differentiation in adipocytes, osteoblasts [3], neural progenitor-like cells [4] and cardiomyocytes [5], under specific conditions. Besides showing dedifferentiation properties, reversine is a kinase inhibitor able to interfere with the growth of several solid tumors, by specifically inhibiting the G2/M checkpoint and cytokinesis. Reversine is an inhibitor of Aurora kinases, Cyclin Dependent Kinases (CDK) and Monopolar Spindle 1 (Mps1), proteins which have a crucial role in mitotic regulation. Aurora-A kinase is a Ser/Thr kinase that affects centrosomes maturation, mitotic entry, centrosome separation, spindle formation and cytokinesis [6]. Aurora-B kinase can modulate various tasks during mitosis, including chromosome migration to the spindle equator, chromosome segregation and cytokinesis [7]. Mps1 is involved in the regulation of spindle checkpoint in part by acting at the level of CDK1-cyclinB complex [8], therefore contributing to the proper attachment of chromosomes to the spindle microtubules before chromosome separation. Interestingly, this protein has emerged as a potential target for cancer therapy and reversine is known to inhibit Mps1 more efficiently than Aurora-B kinase, based on experimental and computational approaches [9]. As inhibitor of Aurora-A and Aurora-B kinases and Mps1, reversine causes an incorrect mitotic spindle assembly and chromosome separation, cell cycle arrest in G2/M phase, endoreduplication and eventually cell death due to a phenomenon called “mitotic catastrophe” observed in many cancer cell lines [10,11,12,13,14,15,16]. Moreover, reversine treatment of human follicular thyroid cancer cells leads to the activation of autophagy due to its capability to down-regulate mTOR [17]. Very recently, reversine has been reported to significantly inhibit the growth of urothelial carcinoma cell lines through activation of AMP activated protein kinase pathway, leading to cell death [18].

The transcription factor p53 is described as the guardian of the genome because it is implicated in several important cell functions, such as DNA repair, cell cycle regulation and apoptosis, preventing tumor formation. In normal conditions the p53 signaling pathway is in standby mode, but in response to cellular stresses p53 is activated and through its sequence-specific DNA binding activity it mediates the transcription of a large number of target genes. p53 is the most commonly mutated gene in many types of human cancers, losing in this way its tumor suppressor properties. Loss of heterozygosity (LOH) is also a frequent event resulting in complete lack of wild type p53 expression in cancer cells. In breast cancer numerous p53 coding mutations were identified, even if the frequency of p53 mutation in this cancer varies among subtypes and overall is approximately at 20% [19]. p53 has been observed to play an important role in modulation of reversine effect. It has been demonstrated that silencing of p53, and the consequent loss of transcriptional activation of its target p21, whose checkpoint function can prevent endoreduplication, renders fibroblasts more sensitive to reversine [10].

A structure activity relationship (SAR) study on a series of reversine-like molecules showing different substitutions on both C-6 purine and C-2 piperazine moieties was carried out with the aim to optimize biological activity, selectivity and oral bioavailability [20]. We report here three 2,6-diamino substituted purine derivatives, selected by docking calculation focused on Aurora-B kinase and Mps1 inhibition, which present the insertion of a new *N*-heterocycle unit in the C-2 substitution and a *N*-methyl group in C-6 unit, enhancing the structural diversity of reversine analogues. In particular, we focused on DFT calculations applied to the first structural investigation of the prototropic tautomers of one of these molecules. The effect of molecules **1**–**3** has been also compared to reversine on breast and colorectal cancer cell lines. We observed that they promoted aberrant mitosis and endoreduplication leading to cell death, possibly via mitotic catastrophe. Moreover, the compounds had a stronger effect when p53 function was ablated.

## 2. Results and Discussion

### 2.1. Selection and Synthesis of Compounds ***1***–***3***

**Figure 1:**
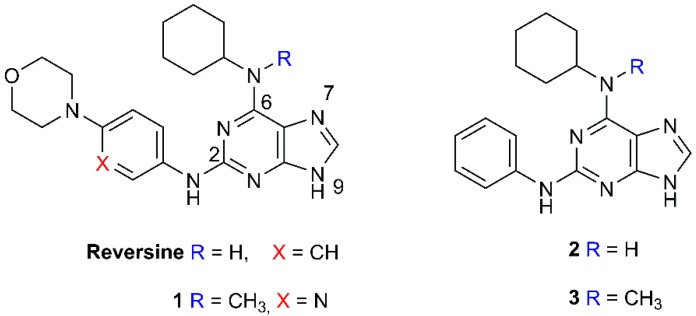
Molecular structures of reversine and synthetic compounds **1**–**3**.

The design of the molecules **1**–**3** (Figure 1) was performed via structure selection based on docking calculations, considering both energy values and interactions of the complexes between the molecules and the two known target proteins of reversine, Aurora-B kinase and Mps1. The results by Autodock Vina indicated that compound **1**, which preserves the reversine skeleton, and the structurally simplified molecules **2** and **3** gave complexes with very similar energy values to those of reversine (Table S1).

**Scheme 1:**
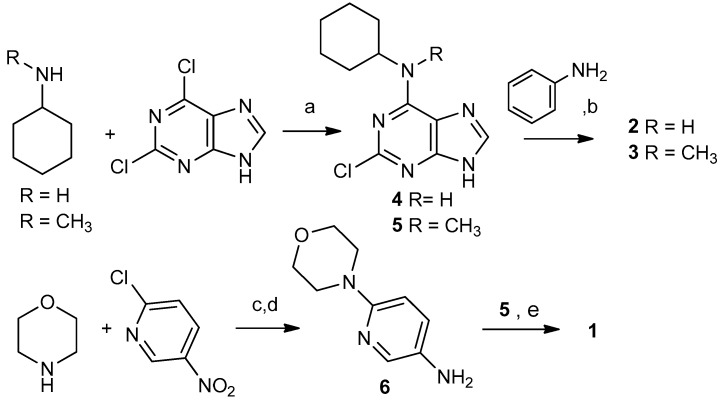
Synthesis of molecules **1**–**3**. *Reagents and conditions*: (**a**) ethanol, TEA, reflux 75 °C, 14 h; (**b**) *N*-methyl-2-pyrrolidone, reflux 150 °C, 14 h; (**c**) DCM, TEA, 12 h; (**d**) H_2_, Pd/C, 2 h; (**e**) ethanol, TFA, Microwave 120 °C, 2.30 h.

Products **2** [21] and **3** were obtained by reacting aniline with **4** and **5**, respectively, the latter were derived by nucleophilic substitution of cyclohexylamine or methylcyclohexylamine with 2,6-dichloropurine (Scheme 1). The production of **5** proceeded with higher yields and in a cleaner way compared to **4**. In fact the methyl-*N*6 substituent as in **5** prevented the formation of the by-product, resulting by the substitution of a second unit of 2,6-dichloropurine at the *N*6 position, as observed in the synthesis of product **4**. The synthesis of **1** was challenging due to the poor nucleophilicity of the aryl amine **6**. Different reaction conditions were compared (Buchwald-Hartwing amination, butanol in the presence of trimethylsilyl chloride or triethylamine; *N*-methyl-2-pyrrolidone (NMP), 150 °C, *p*-toluenesulfonic acid monohydrate in DMSO, trifluoroacetic acid (TFA) in EtOH) under conventional heating or microwave (MW) irradiation, as well as different leaving groups (chlorine as in **5** or a fluorine analogue obtained by 6-chloro-2-fluoro-9*H*-purine). The MW irradiation of **5** and **6** in ethanol in the presence of TFA proved to be the best procedure, although still giving a poor yield in line with data for reactions using similar nucleophiles [21].

The obtained products were characterized by spectrometric measurements including ESI-MS/MS and high resolution experiments by EI-MS technique, and by extensive NMR analysis (see Materials and Methods and Supplementary Material). Prior to their biological evaluation, compound **1** was purified by preparative RP-18 HPLC and the purity of **2** and **3** was evaluated using silica gel HPLC (Materials and Methods).

### 2.2. Study on the Tautomeric Forms of Product ***3***

Both ^1^H- and ^13^C-NMR spectra of products **1** and **3** recorded in CDCl_3_ solution at room temperature showed very broad H_3_C-*N* and HC-*N* signals (Figures S1 and S3), a behavior also observed for the precursor **5**. The broad nature of these signals would normally suggest the presence of tautomers and/or restricted conformations. This is in line with the report of coexisting tautomers of purine bases, with the prototropic H*N*(7) and H*N*(9) as the preferred species, where the intramolecular proton-transfer is accompanied by migration of π electrons. In general their relative populations could be affected by structural factors such as aromaticity, intramolecular interactions, substituent effects able to modify the electronic distribution within the molecule, repulsions of lone electron pairs and also by external influences such as solvent and temperature [22].

In order to gather information on the effective structures of the molecule and to provide details on the interactions involved with biological targets, we decided to further investigate the tautomeric forms of **3**. Thanls to MarvinSketch software analysis the two most probable tautomers were predicted to be the prototropic H*N*(7) and H*N*(9) forms. Firstly, a series of conformations for each tautomer were evaluated by Density Functional Theory (DFT) calculation [22] at a B3LYP/6-31G(d,p) level of theory in the gas-phase, obtaining the corresponding energy values and indications on the energy-minimized structures for the two prototropic forms. In both cases the most stable conformers displayed an *anti*-position of the phenyl-*N*(2) and cyclohexyl-*N*(6) groups (Table 1, entries 1 and 8), with the H*N*(9) structure as the favored one (E = −1027.75607832 a.u.). On the contrary, the structure associated to the highest energy value is the *syn*-H*N*(7) tautomer (Table 1, entry 7). These predictions are in agreement with H*N*(9) tautomer being more favored than H*N*(7), as reported for neutral purine in the gas phase and in non-polar solvents [23], due to the possibility of a stabilizing intramolecular interaction between the hydrogen at *N*(9) position and the lone electron pair on the *N*(1) atom. Besides, the energy minimized structure reported in entry 1 displayed a length of 1.36139 Å for the C(6)-*N* bond, a value which is really intermediate between 1.47357 Å calculated for a single C-N distance and 1.26715 Å for a double C=N distance, indicating a restricted rotation around this bond.

Afterwards, the conformations of the two tautomers were also evaluated in chloroform since it is the solvent used in NMR analysis. The most stable structure (Table 1, entry 1; E = −1027.76438045 a.u.) presented the same conformation as obtained from calculation in the gas phase. Moreover, all the conformations considered for the H*N*(9) tautomer were much more stable than the corresponding ones for the H*N*(7) isomer. Notably, the latter result is in line with both the detections of a single tautomer by NMR spectra and with the very similar energy values for the *anti*- and *syn*-H*N*(9) tautomer obtained by rotation around the *N*-C(6) bond (entries 1 and 3, respectively). These data support their coexistence in solution, responsible for the very broad signals attributable to *N*-CH_3_ group (δ_H_ 3.39 and δ_C_ 30.1 ppm), to the cyclohexylmethine carbon (δ_C_ 54.8 ppm) and to C(6) (δ_C_ 152.3 ppm).

The evaluation of the predominant structure of a ligand molecule present in solution is important in theoretical drug design. It is known that a more biologically relevant water-phase environment can significantly change the relative stability of various tautomers if compared with the gas phase condition [23]. DFT calculations were thus performed in water for the H*N*(9) and H*N*(7) forms of **3** by using the Polarized Continuum Model (PCM). The data reported in Table 1 suggest a similar behavior for the analyzed species both in gas phase and in chloroform solution: the H*N*(9) tautomer resulted more stable than the H*N*(7), with the most stable conformation showing a *syn*-position of the phenyl-*N*(2) and cyclohexyl-*N*(6) groups (E = −1027.76745565 a.u.), very close in energy to the one displaying the *anti*-position (Table 1, entries 3 and 4, respectively).

To gain insight into the probable structural behavior of these molecules involved in interactions with the targets, docking calculations were carried out on both the prototropic forms (Table S1). The results obtained for **3** were in line with the higher stability of H*N*(9) than H*N*(7) tautomer derived by DFT calculations. In both the Aurora-B kinase and Monopolar spindle 1 complexes, H*N*(9) tautomer emerged as the most stable, combining the evaluation of energy data and both H-bonds and hydrophobic interactions with the enzymes. The same trend was observed for molecule **1**. In the case of **2** slightly lower energy values were obtained for H*N*(9) tautomer, but more interactions were associated to H*N*(7) isomer.

### 2.3. Biological Evaluation

We evaluated the growth inhibition effect of the synthetic products in comparison to reversine on two cancer cell lines: the MCF-7 breast cancer (MCF-7 Vector) line and the HCT116 colorectal cancer line. In parallel, we tested whether the effect of reversine and/or molecules **1**–**3** was influenced by the presence of p53 by analyzing their effects both on MCF-7shp53 cell line, stably transfected with a short harpin RNA against p53, and the HCT116p53^−/−^ cells, in which p53 was eliminated by homologous recombination [24,25]. As already demonstrated both cell lines, showed severely reduced or completely abolished levels of full length p53 (Figure S4) [26]. We evaluated the growth inhibition effects by MTT (3-[4,5-dimethylthiazol-2-yl]-2,5 diphenyl tetrazolium bromide) assay after 24 h of treatment, using DMSO (0.1%) as negative control and reversine as positive control (Figure S5). All the compounds **1**–**3** suppressed the growth of MCF-7 and HCT116 cells in a dose-dependent manner. It was evident that their cytotoxicity was similar in the different cancer cell lines, even though HCT116 cells showed a higher resistance to the treatments than MCF-7 cells. Based on MTT results, we calculated the EC_50_ values of reversine and molecules **1**–**3** in MCF-7 and HCT116 cell lines (Table 2). The data demonstrate that these molecules were less toxic than reversine and that EC_50_ indexes were similar for p53 wild-type and p53 compromised cells.

**Figure 2:**
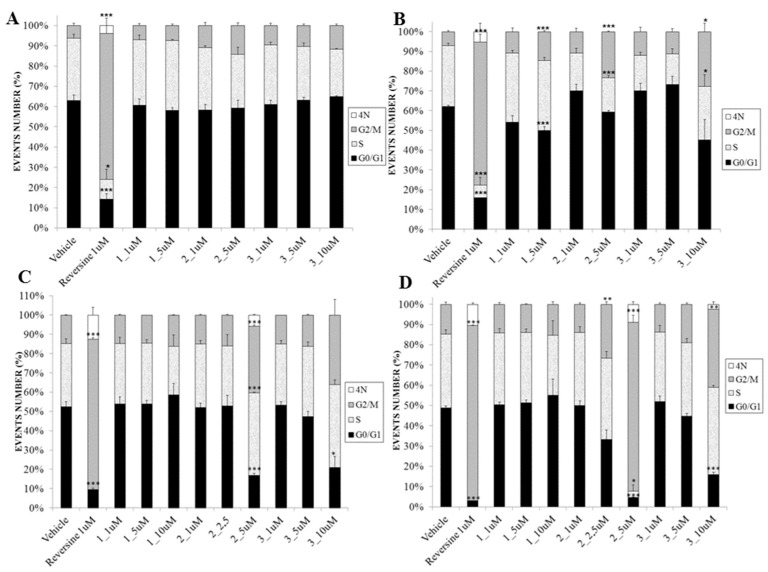
Cell cycle analysis after treatments with compounds **1**–**3** at different concentrations on: (**A**) MCF-7 Vector; (**B**) MCF-7shp53; (**C**) HCT116p53^+/+^ and (**D**) HCT116p53^−/−^. The results are expressed as mean ± SEM of three independent experiments (* *p*-value < 0.05; ** *p*-value < 0.01; *** *p*-value < 0.005).

We next investigated the impact of the products **1**–**3** on cell cycle by flow cytometry. The compounds were able to elicit cell cycle arrest in G2/M phase in a dose-dependent manner, particularly when p53 was compromised. Specifically, after 24 h of treatment, compound **1** and **2** at 5 µM concentration and compound **3** at 10 µM concentration caused a significant cell cycle arrest in MCF-7shp53, but not in MCF-7 vector. The data obtained were statistically significant, as shown by the merge of three experiments (Figure 2A,B). Similar results were obtained in HCT116 cells, even though these cells showed a stronger cycle arrest than MCF-7. Specifically, the fraction of HCT116 cells arrested in the G2/M phase upon treatment with the same dosages used on breast cancer cells (5 µM of **2** and 10 µM of **3**), was 95.22% and 39.63% respectively (Figures S6 and S7). Furthermore, at these concentrations a new peak corresponding to polyploid cells appeared, probably originating from endo-reduplication, an effect caused by the inhibition of mitotic spindle Aurora-A and Aurora-B kinases, and Mps1 [12,14]. The data obtained in triplicates were statistically significant, as shown in Figure 2C,D. The comparison between the effects of analogues **2** and **3** allows us to speculate on the role of the methyl group in C6 position, which represents the only structural difference between the two compounds. Based on the obtained data, the presence of a methyl group in the structure of **3** decreased the efficacy of the molecule. This suggests that the C6-methyl group is responsible for a different and possibly weaker interaction between Aurora kinases and the compound.

**Figure 3:**
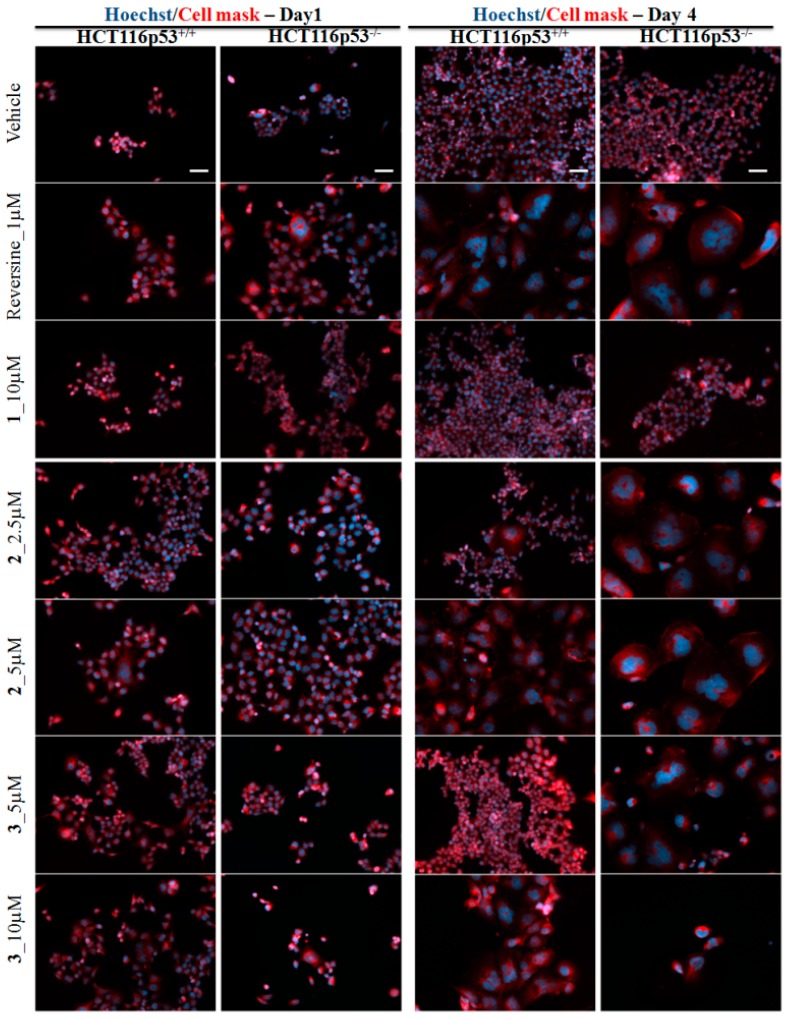
Nuclei and cell membrane staining on HCT116p53^+/+^ and HCT116p53^−/−^ after compounds **1**–**3** treatments at day 1 and day 4 (Scale bar = 50 µm).

A plasma membrane staining was then performed to analyze the effect of the molecules at a morphological level. MCF-7 and HCT116 cell lines were stained and analyzed by fluorescence microscopy upon one and four days of treatment with reversine and **1**–**3** (0.1% DMSO was used as control). Treated MCF-7 cells were larger in size and their number was lower with respect to the control. Furthermore, as shown by 4′,6-diamidino-2-phenylindole (DAPI) staining, many cells presented several nuclei, fused together (Figure S8). This is probably due to the inability to form two separate nuclei during M phase of the cell cycle. Cells could then bypass the G2/M checkpoint by avoiding cell division, therefore generating two fused nuclei inside the same cell. This loop then continues until the cells undergo apoptosis [27,28]. In accordance with data obtained by flow cytometry, this effect was higher in MCF-7shp53 with respect to MCF-7 control cells. Specifically, compound **2** at a 5 µM concentration caused a stronger effect when p53 was down-regulated, while compound **3** at a 10 µM concentration caused cell cycle arrest and endoreduplication only in MCF-7shp53 cells. This result provides an additional confirmation of cell cycle arrest in G2/M phase, probably caused by incorrect mitotic spindle formation and chromosome segregation. Accordingly, similar effects were obtained in HCT116 cells. Specifically, 2.5 µM of **2** and 5 µM of **3** caused cell cycle arrest and endoreduplication in HCT116p53^−/−^ cells but not in HCT116p53^+/+^ cells, with morphological changes more visible at day 4 with respect to day1 (Figure 3).

**Figure 4:**
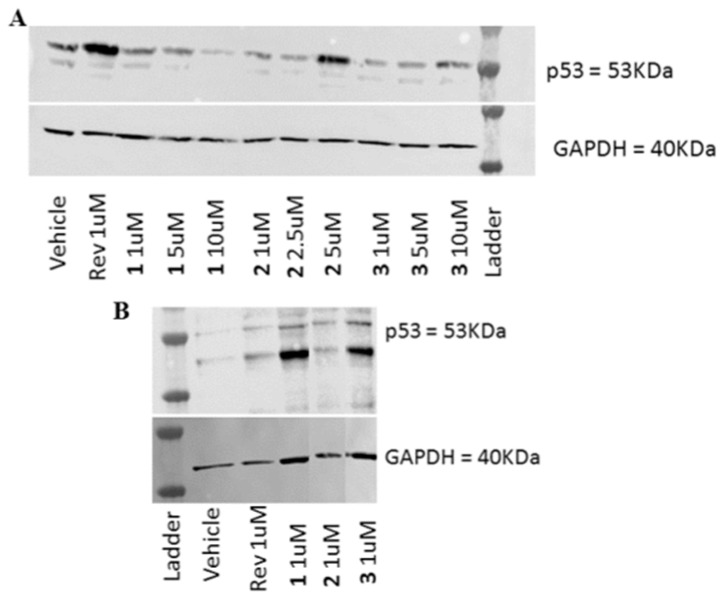
Western blot analysis to determine p53 expression after one day of treatment with different concentration of reversine and compounds **1**–**3** on A MCF-7 Vector and B HCT116p53^+/+^.

The tested molecules are able to stabilize p53 in MCF-7 vector cells at the same concentrations that cause cell cycle aberrations in MCF7shp53. HCT116p53^+/+^ showed upregulation of p53 after treatment with 1 µM reversine as well as with the products **1**–**3** at 1 µM dose, a concentration lower than the lowest effective dose observed in the previous experiments (Figure 4). Our data support the idea that p53 plays a regulatory role in mitotic spindle formation and control of chromosome segregation. p53 is stabilized in response to cellular damage, it accumulates, triggers a reversible G2 arrest and can activate the transcription of genes able to restore microtubules function. Breast cancer 1 (BRCA1) and DNA repair complex proteins could be implicated in this process [27]. Consistent with this view, in wild-type cells p53 could orchestrate damage repair to the mitotic spindle caused by reversine and **1**–**3** so that cell cycle is not strongly affected. Interestingly, p53-null cells treated with a molecule that causes a low genotoxic stress undergo endoreduplication and then die from apoptosis [28].

The 3D structures of the H*N*(7) and H*N*(9) tautomers of **1**–**3**, obtained by docking calculation with both kinases have been analyzed in detail. In the complexes with both kinases, for **1** and **3**, it was evident that Me-*N*-C6 group is located inside the active center with no steric hindrance and is not involved in any interactions. In particular, regarding the complexes of Aurora B (2VGO) with the H*N*(9) tautomers, a good overlapping of the structures of reversine and of **1**–**3** becomes particularly evident, but the *N*-cyclohexyl unit takes on the same disposition for reversine and **2**, differently from **1** and **3** (Figure S9). Moreover, a different disposition is evident for the H*N*(7) tautomers of **2** and **3** in the complexes, as evident in Figure S10. Notably, in the docking calculation of **2** with both Aurora-B kinase and Monopolar Spindle 1 (Table S1), the energy differences between H*N*(9) and H*N*(7) tautomers resulted lower than the corresponding values obtained for **1** and **3**. This suggest a favored condition for the H*N*(7) isomer in **2** due to the absence of the *N*-methyl C(6) group. Moreover, the rotation around the *N*-C(6) bond may increase the conformations of this isomer, differently from the restricted rotation in **3** and **1**. These computational data support the higher kinase inhibition by molecule **2** than **3** and **1**.

## 3. Materials and Methods

### 3.1. Chemistry

#### 3.1.1. General

Reversine and all reagents were purchased from Sigma Aldrich (St. Louis, MO, USA) and used without further purification. The reaction yields were calculated for the products after chromatographic purification. Thin layer chromatography (TLC): Merck silica gel F_254_ or reversed phase Merck RP-18 F_254_ (Merck, Darmstadt, Germany), with visualization with UV light. Flash chromatography (FC): Merck Si 15–25 μm; preparative thin layer chromatography (PLC): 20 × 20 cm Merck Kieselgel 60 F_254_ 0.5 mm plates. MW-assisted reactions were carried out using a mono-mode CEM Discover SP reactor (CEM, Matthews, NC, USA) in a sealed vessel (200 W max). NMR spectra were recorded on an Avance 400 spectrometer (Bruker, Billerica, MA, USA) using a 5 mm BBI probe ^1^H at 400 MHz and ^13^C at 100 MHz in CDCl_3_ (relative to δ_H_ 7.25 and δ_C_ 77.00 ppm), or in (CD_3_)_2_SO (relative to δ_H_ 2.50 and δ_C_ 39.50 ppm), δ values in ppm, *J* values in Hz; assignments are supported by heteronuclear single quantum correlation (HSQC) and heteronuclear multiple bond correlation (HMBC) experiments. Electrospray ionization (ESI)-MS mass spectra were recorded using a Bruker Esquire-LC spectrometer by direct infusion of a methanol solution (source temperature 300 °C, drying gas N_2_, 4 L/min, scan range *m*/*z* 100–1000). Electron ionization (EI) mass spectra (*m*/*z*; rel%) and high resolution (HR)-EI data were recorded with a Kratos-MS80 mass spectrometer (SIS, Ringoes, NJ, USA), heating at 213 °C for **2**, at 276 °C for **1** and at 417 °C for **3**.

#### 3.1.2. Typical Reaction Procedure for Precursors **4** and **5**

To a solution of 2,6-dichloropurine (260 mg, 1.38 mmol, 1.0 equiv) in EtOH (2 mL) cyclohexylamine or methylcyclohexylamine (1.52 mmol, 1.1 equiv) and Et_3_N (1.52 mmol, 1.1 equiv) were added. The reaction mixture was irradiated at 90 °C for 1.5 h in a microwave reactor. The obtained white precipitate was filtered and chromatographically purified under the indicated conditions.

*2-Chloro-N-cyclohexyl-9H-purin-6-amine* (**4**). TLC (CH_2_Cl_2_:MeOH = 94:6 *v*/*v*): R_f_ = 0.55. Purification by silica gel PLC (CH_2_Cl_2_:MeOH = 94:6 *v*/*v*) Yield: 65%. ^1^H-NMR (DMSO-*d*_6_) δ 13.05 (br s, 2H, NH_2_), 8.08 (s, 1H), 7.95 (brs, 1H, NH), 4.60 (br s), 3.96 (br s cyclohexyl CH), 1.89, 1.73 and 1.26 (series of m, 10H, cyclohexyl). ^13^C-NMR (DMSO-d_6_) δ 154.2, 152.9, 150.5, 139.2(N=CH), 117.8, 48.8 (cyclohexyl CH), 32.1 (2C), 25.1 (2C), 24.8. Significant HMBC correlation: 1.26 ppm with 48.8 ppm. ESI(−)-MS: *m*/*z* 250/252 in ca. 3:1 ratio, [M − H]^−^.

*2-Chloro-N-cyclohexyl-N-methyl-9H-purin-6-amine* (**5**). TLC (hexane: EtOAc = 4:6 *v*/*v*): R_f_ = 0.62. Purification by silica gel FC (hexane:EtOAc, from 7:3 to 4:6 *v*/*v*). Yield: 80%. ^1^H-NMR (CDCl_3_) δ 13.30 (br s, 1H, *N*H), 7.84 (s, 1H, H=CN), 4.88(br s, 1H, cyclohexyl CH), 3.18 (br s, 3H, *N*CH_3_), 1.86, 1.73, 1.17 (series of m, 10H, cyclohexyl). ^13^C-NMR (DMSO-*d*_6_, detectable signals) δ 154.5, 152.9, 138.7(HC=N), 118.3 (br), 25.7(2C), 25.4. ESI(−)-MS: *m*/*z* 264/266 in 3:1 ratio, [M − H]^−^.

#### 3.1.3. Synthesis of 6-Morpholinopyridin-3-amine (**6**)

A mixture of 2-chloro-5-nitropyridine (303 mg, 1.91 mmol, 1.0 equiv), morpholine (0.5 mL, 5.74 mmol, 3.0 equiv) and Et_3_N (483 mg, 0.67 mL, 2.5 equiv) in CH_2_Cl_2_ (4 mL) was stirred at room temperature overnight. The reaction mixture was diluted with water (10 mL) and extracted with CH_2_Cl_2_ (30 mL × 3). The combined organic layers were washed with water (30 mL × 6) and brine (1 × 30 mL), dried over anhydrous Na_2_SO_4_ and concentrated in vacuo to give a yellow solid. 70 mg of the yellow solid (0.335 mmol, 1.0 eq) were diluted in EtOH (5 mL) and a spatula tip of catalyst Pd/C was added. The obtained mixture was hydrogenated for 2 h, using a Hypem XP hydrogen generator (h2planet, Milan, Italy), Pressure was set at 1.5 bar. The crude mixture was filtered on Celite, and the filtrate was evaporated to obtain a red solid. Yield: 93% over two steps. TLC (hexane:ethyl acetate = 4:6 *v*/*v* + Et_3_N): R_f_ = 0.15. ^1^H-NMR (CDCl_3_) δ 7.79 (d, *J* = 2.7 Hz, 1H), 7.01 (dd, *J* = 8.8, 2.7 Hz, 1H), 6.73 (brs, 2H, NH_2_), 6.56 (d, *J* = 8.8 Hz, 1H), 3.82 (m, 4H), 3.33 (m, 4H). ^13^C-NMR (CDCl_3_) δ 154.02, 135.09, 134.58, 126.42, 108.41, 66.80 (2C), 47.08 (2C). ESI(−)MS: *m*/*z* 178 [M − H]^−^.

#### 3.1.4. Synthesis of *N*6-Cyclohexyl-*N*6-methyl-*N*2-(6-morpholinopyridin-3-yl)-7*H*-purine-2,6-diamine (**1**)

A mixture of **5** (22.5 mg, 0.085 mmol, 1 equiv), **6** (60 mg, 0,34 mmol, 4 equiv) and trifluoroacetic acid (0.1 equiv.), in ethanol (2 mL), was microwave irradiated at 120 °C, for 2.5 h. The reaction mixture was concentrated in vacuo and the residue was submitted to preparative HPLC (RP-18 LiChroSphere 7 µm, acetonitrile/water 1:1 + 0.1% TFA, flow 5 mL/min, detection at 268 nm, 3.1 min) obtaining **1** trifluoracetate salt as a white powder (10.7 mg, yield 25%). ^1^H-NMR (CDCl_3_) δ 12.40 (br s, 1H), 7.94 (brd, *J* = 7.8 Hz, 1H), 7.83 (s, 1H), 7.79 (brs, 1H), 7.03 (brd, *J* = 8.1 Hz, 1H), 6.57 (brd, *J* = 8.1 Hz, 1H), 3.82 (m, 4H), 3.35 (m, 4H), 3.20 (brs, NCH_3_), 2.16–1.28 (series of m, 10H). ^13^C-NMR (CDCl_3_) detectable signals by HSQC correlation δ 7.83 with δ 135.7 (C-8) and by HMBC correlations: δ 53.6, 151.9, 118.4, 133.6, 127.0, 108.6, 66.2, 46.6. ESI(+)-MS: *m*/*z* 409 [M + H]^+^; MS/MS (409): *m*/*z* 327. The solid, dissolved in methanol, was treated with Et_3_N, the mixture evaporated in vacuo and the residue eluted through RP-18 LiChrolut with water/methanol, gradient elution to obtain free **1**. ESI(−)MS: *m*/*z* 407 [M − H]^−^; MS/MS(407): *m*/*z* 325. EI-MS: *m*/*z* 408 (M^+^, 5), 368 (4), 326 (1), 229 (2), 179 (23). HRMS(EI) calcd. for C_21_H_28_N_8_O, 408.23861, found 408.23683.

#### 3.1.5. Typical Reaction Procedure for Products **2** and **3**

Compound **4** or **5** (0.188 mmol, 1.0 equiv.) was dissolved in *N*-methyl-2-pyrrolidone and aniline (0.56 mmol, 3.0 equiv.) was added. The reaction mixture was heated at 150 °C for 14 h, then ice-water (15 mL) was added and the mixture was extracted with EtOAc (20 mL × 2). The combined organic layers were washed with ice-water mixture (15 mL × 5) and brine (20 mL), dried over anhydrous MgSO_4_, and concentrated under reduced pressure to give a brown syrup. The crude residue was purified by silica gel FC eluting with hexane/EtOAc (from 7:3 to 4:6 *v*/*v*). The purity of **2** and **3** was verified by analytical HPLC injection (Si60 LiChroSphere 15–25 µm, 254 nm) with hexane/2-propanol 9:1.

*N*6-Cyclohexyl-*N*2-phenyl-7*H*-purine-2,6-diamine (**2**). TLC (EtOAc): R_f_ = 0.42. Yield: 70%. ^1^H-NMR (CDCl_3_) δ 13.0 (br. s., 1H, NH), 7.51 (brd., *J* = 7.8 Hz, 2H), 7.31 (brt, *J* = 7.8 Hz, 2H), 7.05 (brt, *J* = 7.8 Hz, 1H), 7.03 (s. 1H, purine), 6.57 (s, 1H, NH, exchangeable by CD_3_OD addition), 5.59 and 4.01 (1:1 two brs, 1H, NH), 2.05 (m, 1H), 1.74 (m, 2H), 1.64 (m, 2H), 1.33 (m, 6H), in agreement with reported data [21]. ^13^C-NMR (CDCl_3_) δ 156.6, 154.4, 150.3 (v br), 139.9, 135.9, 129.2 (2C), 123.0, 121.2 (2C), 114.7 (br), 49.3 (v br), 33.3 (2C), 25.6, 24.9 (2C). ESI(+)-MS: *m*/*z* 309 [M + H]^+^; MS/MS (309): *m*/*z* 227. EI-MS: *m*/*z* 308 (M^+^, 100), 225 (67). HRMS(EI) calcd. for C_17_H_20_N_6_, 308.17494, found 308.17510.

*N*6-Cyclohexyl-*N*6-methyl-*N*2-phenyl-7*H*-purine-2,6-diamine (**3**). TLC (EtOAc): R_f_ = 0.54. Yield: 74%. ^1^H-NMR (CDCl_3_) δ 12.61 (brs., 1H, NH), 7.54 (brd, *J* = 7.8 Hz, 2H), 7.31 (brt, *J* = 7.8 Hz, 2H), 7.04 (brt, *J* = 7.8 Hz, 1H), 6.92 (s., 1H, NH, exchangeable), 6.77 (s, 1H, purine), 5.17 (br s, 1H, NH), 3.39 (br s, 3H, NCH_3_), 1.90–1.12 (series of m, 10H). ^13^C-NMR (CDCl_3_) δ 154.8, 155.6, 152.3 (br), 140.2, 134.6, 129.1 (2C), 122.6, 120.6 (2C), 114.7 (br), 54.8 (very br), 30.1 (br, CH_3_), 25.7 and 25.8 (5C). ESI(+)-MS: *m*/*z* 323 [M + H]^+^; MS/MS (323): *m*/*z* 241 EI-MS: *m*/*z* 322 (M^+^, 95), 307 (50), 265 (56), 240 (72). HRMS(EI) calcd. for C_18_H_22_N_6_, 322.19059, found 322.19071.

### 3.2. Computational Analysis

DFT calculation was performed for the tautomers of **3** in the gas phase, in chloroform and in water by using Polarized Continuum Model (PCM). Calculations were carried out on a PC running at 3.4 GHz on an Intel i7 2600 quad core processor with 8 GB RAM and 1 TB hard disk with Windows 7 Home Premium 64-bit SP1 as an operating system. Ligands were build using PC Model version 6.0 (Serena Software, Bloomington, IN, USA). A Gaussian 03W revision E.01 program [29] with graphical interface GaussView 4.0. was used in the geometry optimization at a density functional theory (DFT) level of theory and invoking gradient with 6-31G(d,p) basis set for all the atoms. The gradient-corrected DFT with the three-parameter hybrid functional (B3) [30] for the exchange part and the Lee–Yang–Parr (LYP) correlation function [31] were utilized. The optimized structural parameters were employed in the vibrational energy calculations at the DFT levels to characterize all stationary points as minima. No imaginary wave number modes were obtained for each optimized structure, proving that a local minimum on the potential energy surface was actually found. The minimized molecules were saved in pdb extension.

AutoDock Tools (ADT) package version 1.5.6rc3 was employed to generate the docking input files and to analyze docking results, whereas Autodock Vina 1.1.2 [32] was taken for docking calculation. The structures of Aurora-B (PDB ID: 2VGO) and Mps1 (PDB ID: 3HF9) kinases were determined by X-ray crystallography with a resolution of 1.7 Å and 2.6 Å respectively [12,33]. The structures were modified as follows: the ligand and all the crystallization water molecules were removed saving the file in pdb extension. All the hydrogen atoms were added by AutoDock Tools (ADT) and Gasteiger–Marsili charges were calculated saving the resulting file in pdbqt extension. Rotatable bonds were defined for each minimized ligand molecule. For the docking calculation a grid box of 16 × 16 × 16 Å in *x*, *y*, *z* directions was created, spacing of 1.00 Å and centered at *x* = 10.52, *y* = −0.323, *z* = 3.0766 for 2VGO and a grid box of 14 × 14 × 14 Å in *x*, *y*, *z* directions was created, spacing of 1.00 Å and centered at *x* = 0.572, *y* = 17.486, *z* = 51.185 for 3H9F. Vina parameters were set as follows: exhaustiveness of the local search =100 and number of conformations to calculate =10. Results are expressed as energy associated to each ligand-enzyme complex in terms of Gibbs free energy values (Table S1). The visual ligand-enzyme interactions were displayed using LigPlot [34].

### 3.3. Biological Evaluation

#### 3.3.1. General

The purity of the tested compounds is >99% as established by HPLC analysis. The compounds were dissolved in DMSO. All treatments were conducted 24 h after cell seeding in culture medium.

#### 3.3.2. Cell Culture

p53 wild-type (MCF-7 Vector) and silenced version of MCF-7 (MCF-7 shp53) were received from Reuven Agami [17]. HCT116 p53^+/+^ and the derivative p53^−/−^ cell lines were obtained from Bert Vogelstein [19]. All cell lines were cultured in RPMI pH 7.4 (Thermo Fisher Scientific, Waltham, MA, USA) supplemented with 10% Fetal Bovine Serum (Mill Creek, Rochester, MN, USA), 1X L-Glutamine and Pen/Strep (Lonza, Basel, Switzerland) at 37 °C with 5% CO_2_ in a humidified atmosphere. For MCF-7, 0.5 µg/mL Puromycin (Sigma Aldrich) was added to the culture medium to maintain selection of the vectors.

#### 3.3.3. Cell Viability Assay (MTT Assay)

For the MTT assay, 5 × 10^3^ cells/100 µL were plated into 96-well tissue culture plates. The day after, cells were treated with medium containing 0.1% DMSO alone or medium containing several concentrations of reversine or analogues (0.01 µM, 0.1 µM, 0.5 µM, 1 µM, 5 µM, 10 µM, 50 µM, 100 µM and 300 µM). After 24 h incubation, cell viability was determined by tetrazolium dye 3-(4,5-dimethylthiazol-2yl)-2,5-diphenyl-2H-tetrazolium bromide (MTT) (5 mg/mL in phosphate buffer saline (PBS); Sigma Aldrich) which was added to fresh medium (final concentration: 0.5 mg/mL). The yellow redox indicator MTT is reduced to a dark blue final product, MTT-formazan, by the mitochondrial dehydrogenases of living cells. Following 1 h-incubation at 37 °C, the medium was discarded and cells were lysed by adding 100 µL DMSO. Finally absorbance was examined at 590 nm wavelength using an Infinite M200 microplate reader (TECAN, Mannedorf, Switzerland). Each experiment has been run in triplicate and results are the merge of three experiments.

#### 3.3.4. Cell Cycle Assay

Cell cycle analysis was performed on at least 30,000 events for each sample with FACSCantoTM A using BD Diva Software 6.1.3 (BD Bioscience, San Jose, CA, USA) and the DNA profile was analyzed by ModFit LT 4.1 (Verity Software House, Topsham, ME, USA). Cells were treated with 0.1% DMSO, 1 µM Reversine or different concentrations of each **1**–**3** (1 µM, 2.5 µM, 5 µM and 10 µM) for 24 h, detached with trypsin-EDTA, collected by centrifugation and washed three times with PBS. Cells were stained with propidium iodide using BD CycletestTM Plus DNA Kit (BD Bioscience) and then analyzed by flow cytometry. Results obtained are the merge of three experiments.

#### 3.3.5. Membrane and Nuclei Staining

After treatments, cultured cells were washed twice with PBS and fixed with 4% formaldehyde/PBS for 20 min at room temperature. After three washes in PBS, cells were stained with Hoechst (Thermo Fisher Scientific, Waltham, MA, USA; 1:10,000) and Cell Mask (Thermo Fisher Scientific, Waltham, MA, USA; 1:10,000) and then visualized with a Zeiss Axio Observer Z1 instrument (Carl Zaiss, Oberkochen, Germany) using Zeiss AxioVision v.4.8.1.

#### 3.3.6. Western Blot

Cells were cultured in 10 cm^2^/well and treated with 1 µM reversine or different concentrations of analogues (1 µM, 2.5 µM, 5 µM and 10 µM) for 24 and 96 h. 0.1% DMSO was used as negative control. Cells were detached with trypsin-EDTA, collected by centrifugation and washed with PBS. Samples were then lysed in 150 µL RIPA buffer (Sigma Aldrich) and the proteins were quantified by BCA assay (Thermo Scientific). 15 µg of extracted proteins were loaded on NuPAGETM 4–12% Bis-Tris acrylamide Gel (Thermo Fisher Scientific) and then transferred onto a nitrocellulose membrane using a Tris-Glycine buffer. Blocking was performed overnight with 5% not-fat dry milk, 0.1% TWEENTM and PBS1X. Immunodetection was obtained using the following primary antibodies: p53 (DO1, 1:4000; Santa Cruz Biotechnologies, Dallas, TX, USA) and GAPDH (1:10,000; Santa Cruz Biotechnologies). The secondary antibody used was horse alpha-mouse Human Recombinant Peroxidase (1:10,000; Vector Laboratories, Burlingame, CA, USA). Finally, western blots were analyzed by ECL and detected with ChemiDocTM XRS+ (Bio-Rad, Hercules, CA, USA) using ImageLab software (Bio-Rad).

#### 3.3.7. Statistical Analysis

Data are presented as mean ± SEM of three independent experiments. GraphPad Prism 6 software (GraphPad software, La Jolla, CA, USA) was used and ANOVA test was performed for comparisons. Statistical significance was defined as a *p* value less than 0.05 in all tests.

## 4. Conclusions

Based on the known antitumor effects of reversine, we have selected three structurally related molecules, designed as kinase inhibitors, by a docking calculation approach. They are 2,6-diamino substituted purines, two of them characterized by a simpler structure than reversine and easily accessible by synthesis. A first in vitro evaluation showed that molecules **1**–**3** were less cytotoxic than reversine in breast cancer MCF-7 and colorectal HCT116 cancer cell lines. They caused a significant arrest in G2/M phase in a cell-type dependent mode, as HCT116 cells were more responsive than MCF-7. Moreover, in both cancer cell lines, the molecules showed an interesting increased efficacy when p53 was down-regulated. These results have been confirmed by membrane and nuclei staining. In conclusion, these biological experiments suggest that the presence of a *N*-methyl group in position C6 has a decisive role in affecting the activity of the tested molecules. It is clearly evident comparing the structures of **2** and **3**, where the methyl absence in **2** increases its biological effect requiring a lower concentration to cause the mitotic spindle catastrophe in the cell lines tested. A similar behavior was observed also for reversine and its most structurally similar molecule **1**. Based on the findings in this work and on their easily accessible molecular structures, compounds **2** and **3** are worthy of further biological investigations.

## Figures and Tables

**Table 1:** Minimized structures and energy values of four conformations for each prototropic tautomer H*N*(9) and H*N*(7) of compound **3** (entries 1–4 and 5–8, respectively) evaluated by DFT at level B3LYP/6-31G(d,p) in the gas phase, in chloroform and in water. Numbering positions follow the indication in Figure 1.

Entry	Minimized Structure	Condition	ΔE (kJ/mol)
1	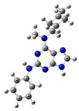	gas phase	--
		CHCl_3_	--
		water	17.58954
2	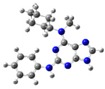	gas phase	6.33039
		CHCl_3_	6.24671
		water	4.35136
3	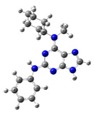	gas phase	1.56063
		CHCl_3_	1.86188
		water	--
4	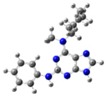	gas phase	3.46017
		CHCl_3_	3.11708
		water	1.058552
5	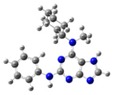	gas phase	4.92875
		CHCl_3_	21.17926
		water	18.43052
6	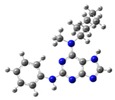	gas phase	6.7195
		CHCl_3_	29.88213
		water	21.50576
7	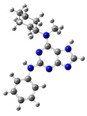	gas phase	35.5933
		CHCl_3_	22.57268
		water	13.31767
8	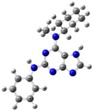	gas phase	2.87441
		CHCl_3_	26.39267
		water	17.58954

**Table 2:** Cytotoxicity values (EC_50_, µM) of reversine and molecules **1**–**3** tested on MCF-7 breast cancer (Vector and p53 downregulated) and HCT116 colorectal cancer (p53^+/+^ and p53^−/−^) cell lines.

Compound	MCF-7 Vector	MCF-7 shp53	HCT116 p53^+/+^	HCT116 p53^−/−^
**Reversine**	9.5	11.0	16.7	7.5
**1**	99.5	111.2	117.2	157.9
**2**	35.3	39.7	40.4	38.1
**3**	35.7	36.5	73.2	67.6

## Supplementary Materials

The following material is available online at http://www.mdpi.com/1420-3049/23/8/1996/s1: computational details; Table S1: Data from docking calculation by Autodock Vina of reversine and the H*N*(7) and H*N*(9) tautomers of each molecule **1**–**3** with Aurora-B Kinase and Monopolar Spindle 1; Figures S1–S3: NMR spectra (CDCl_3_) of compounds **1**–**3**; Figure S4: Western Blot to control the presence or absence of p53 in MCF7 and HCT cell lines. Cells were treated with and without doxorubicin; Figure S5: Data for reversine and **1**–**3** which suppressed the growth of human cancer cells; Figure S6: Representative images from analysis of MCF-7 vector and MCF-7shp53 cell cycle after the treatment with different doses of **1**–**3**; Figure S7: Representative images from analysis of HCT116 wt and HCT116 p53^−/−^ cell cycle after the treatment with different doses of **1**–**3**; Figure S8: Nuclei and cell membrane staining on MCF-7 Vector and MCF-7shp53 after **1**–**3** treatments at day 1 and day 4 (Scale bar = 50 µm); Figure S9: Superimposed structures of H*N*(9) tautomers of reversine (green) and molecules 1(red), 2 (magenta) and 3 (blue) in the complexes with Aurora-B kinase (2VGO), as obtained by docking calculation; Figure S10: Superimposed structures of H*N*(7) tautomers of molecules 2 (magenta) and 3 (blue) in the complexes with Aurora-B kinase (2VGO), as obtained by docking calculation.
