# Supplementary material for: Synthesis of 2,6-Diamino-Substituted Purine Derivatives and Evaluation of Cell Cycle Arrest in Breast and Colorectal Cancer Cells
Source: Molecules. 2018 Aug 10;23(8):1996. doi: 10.3390/molecules23081996 (PMC6222518; doi:10.3390/molecules23081996)
Supplement: Supplementary file 1 [file molecules-23-01996-s001.pdf]

Supplementary Material

Synthesis of 2,6-Diamino-susbtituted Purine Derivatives and Evaluation of Cell Cycle Arrest in Breast and Colorectal Cancer Cells

Bartolomeo Bosco[1], Andrea Defant[2], Andrea Messina[1], Tania Incitti[1], Denise Sighel[1,2], Angela Bozza[1], Yari Ciribilli[1], Alberto Inga[1], Simona Casarosa [1,*] and Ines Mancini [2,*]

[1] Centre for Integrative Biology (CIBIO), University of Trento, Via Sommarive 9, 38123 Trento, Italy;
[2] Laboratory of Bioorganic Chemistry, Department of Physics, University of Trento, Via Sommarive 14, 38123 Trento, Italy;
* Correspondence: ines.mancini@unitn.it; Tel.: +39-461-281-548

Table of Contents

Table S1. Data from docking calculation by Autodock Vina of reversine and the H*N*(7) and H*N*(9) tautomers of each molecule **1-3** with Aurora-B Kinase and Monopolar Spindle 1.

Figure S1. ^{1}HNMR spectrum (400MHz, $CDCl_3$) of reversine analogue **1**, isolated as trifluoroacetate salt.
Figure S2. ^{1}H- and ^{13}CNMR spectra ($CDCl_3$) of compound **2**.
Figure S3. ^{1}H- and ^{13}CNMR spectra ($CDCl_3$) of compound **3**.
Figure S4. Western Blot to control the presence or absence of p53 in MCF7 and HCT cell lines. Cells were treated with and without Doxorubycin, an activator of p53.
Figure S5. Reversine and the related molecules **1-3** suppressed the growth of human cancer cells. A MCF-7 Vector, B MCF-7shp53, C HCT116p53$^{+/+}$ and D HCT116p53$^{-/-}$, were incubated with reversine or its analogues with multiple dosage for 24 hrs. Cell viability was determined by MTT assay. Results are the merge of three experiments.
Figure S6. Representative images from analysis of MCF-7 Vector and MCF-7shp53 cell cycle after the treatment with different doses of compounds **1-3.** After the treatment a propidium iodide staining was performed and analyzed by flow cytometry.
Figure S7. Representative images from analysis of HCT116 wt and HCT116 p53$^{-/-}$ cell cycle after the treatment with different doses of compounds **1-3.** After the treatment a propidium iodide staining was performed and analyzed by flow cytometry.
Figure S8. Nuclei and cell membrane staining on MCF-7 Vector and MCF-7shp53 after **1-3** treatments at day 1 and day 4 (Scale bar = 50μm).
Figure S9. Superimposed structures of H*N*(9) tautomers of reversine (green) and molecules **1**(red), **2** (magenta) and **3** (blue) in the complexes with Aurora-B kinase (2VGO), as obtained by docking calculation.
Figure S10. Superimposed structures of H*N*(7) tautomers of molecules **2** (magenta) and **3** (blue) in the complexes with Aurora-B kinase (2VGO), as obtained by docking calculation.

Table S1. Data from docking calculation by Autodock Vina of reversine and the H*N*(7) and H*N*(9) tautomers of each molecule **1-3** with Aurora-B Kinase and Monopolar Spindle 1.

	Aurora-B kinase (PDB ID: 2VGO)			*Monopolar spindle 1*(PDB ID: 3H9F)		
**Compound**[a]	**ΔE**[b]	**H-bond**[c,d]	**Aminoacidic hydrophobic interactions**	**ΔE**[b]	**H-bond**[c,d]	**Aminoacidic hydrophobic interactions**
**Reversine**[e]	-35.56	C(2)-N/L99 (3.15)	V107, E171, F172, A173, R175, G176, E177, K180, L223	-39.75	N(9)/E603 (2.87)<br>C(2)-N/G605 (3.34)	K529, I531, V539, A551, I586, M602, C604, N606, I607, D608, L654, I663, D664, M671, Q672, P673,
**1a**	-35.98	C(2)-N/L99 (3.16)	V107, E171, F172, A173, R175, G176, E177, K180, L223, A233	-36.40	-	I531, V539, A551, I586, M602, E603, G605, N606, I607, D608, L654, I663, D664, M671, Q672, P673
**1b**	-37.24	C(2)-N/L99 (3.20)	V107, E171, F172, A173, R175, G176, E177, K180, L223, A233	-38.49	N(9)/E603 (2.87)	I531, V539, A551, I586, M602, C604, G605, N606, I607, D608, S611, L654, I663, D664, Q672, P673
**2a**	-32.64	C(6)-N/E177(3.14)	L99, K101, V107, F172, A173, R175, G176, K180, L223	-35.56	N(9)/D664 (3.13)<br>C(2)-N/D608 (3.12)	K528, I531, V539, A551, I586, E603, N606, I607, L654, I663, M671, Q672, P673
**2b**	-33.05	-	L99, V107, F172, A173, G176, E177, K180, L223	-36.82	C(2)-N/G605 (3.32)	I531, V539, A551, I586, M602, E603, C604, N606, I607, D608, L654, I663, D664, M671, Q672, P673
**3a**	-34.31	C(2)-N/L99 (2.87)	V107, F172, A173, G176, E177, K180, L223	-33.47	-	I531, G605, N606, I607, D608, S611, D664, P673, D674 T675
**3b**	-35.56	C(2)-N/L99 (2.97)<br>N(9)/A173 (3.15)	V107, F172, G176, E177, K180, L223, A233	-35.98	N(9)/E603 (3.08)	I531, A551, I586, C604, G605, I607, D608, L654, I663, D664, M671, Q672, P673

[a] **a**=H*N*(7) tautomer; **b**= H*N*(9) tautomer

[b] Calculated docking energy in kJ/mol

[c]Position on the molecular structures in according to the numbering reported in Figure1

[d]H-bond distance in Å reported in brackets

[e] H*N*(9) tautomer according to the crystal structure of reversine-Aurora B kinase complex (D'Alise, A.M. *et al.*, *Mol.Cancer Ther.* **2008**, *7*, 1140-1149. doi:10.1158/1535-7163.MCT-07-2051) cited in file 2VGO.pdb.

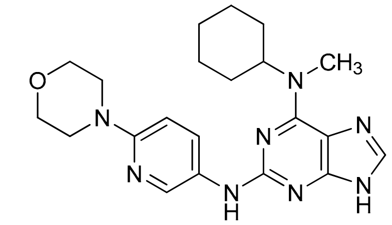

1

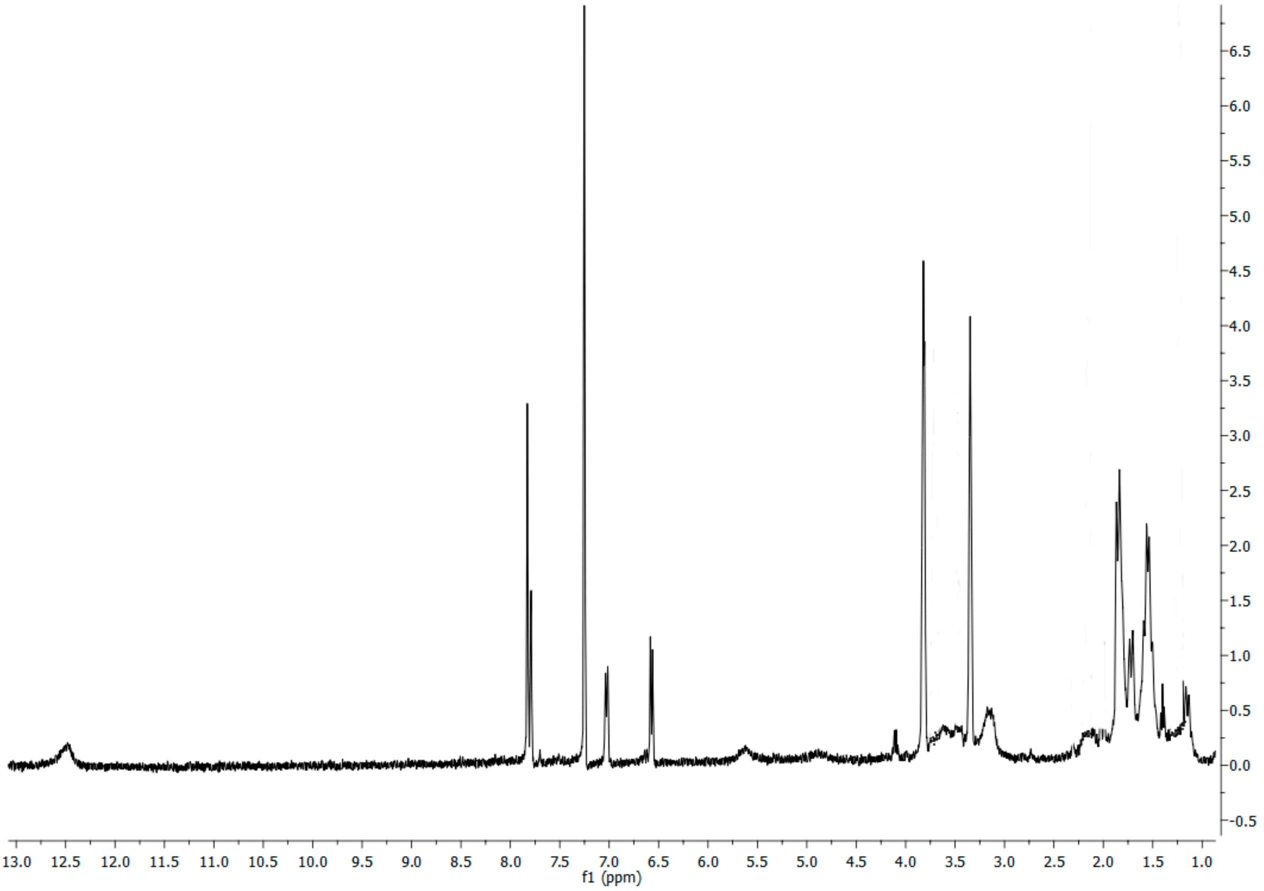

Fig. S1. ^{1}HNMR spectrum (400MHz, $CDCl_3$) of reversine –like molecule **1**, isolated as trifluoroacetate salt.

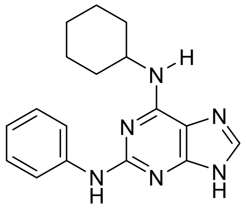

2

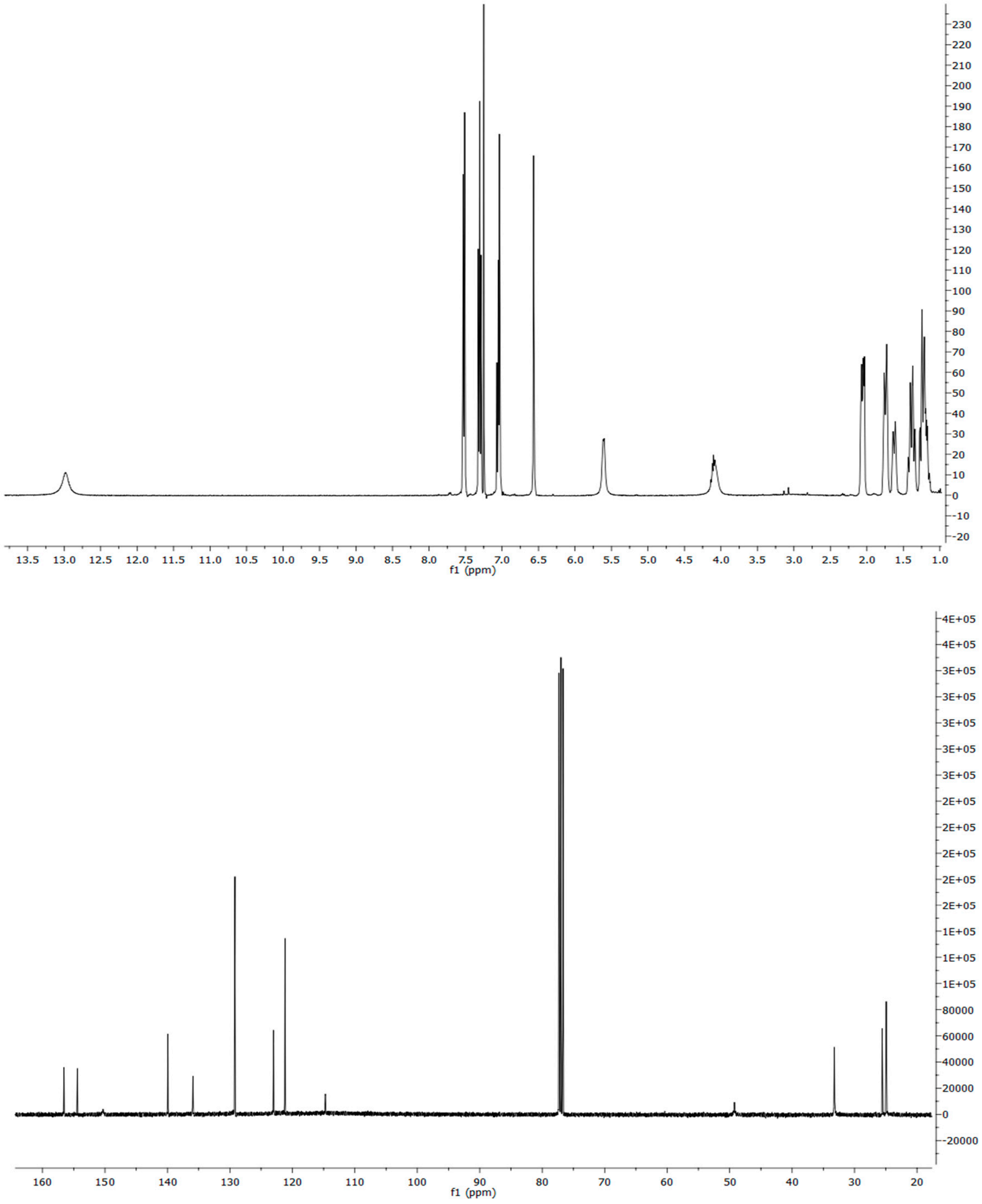

Fig. S2. ^{1}H- and ^{13}CNMR spectra ($CDCl_3$) of compound **2**.

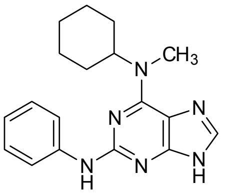

3

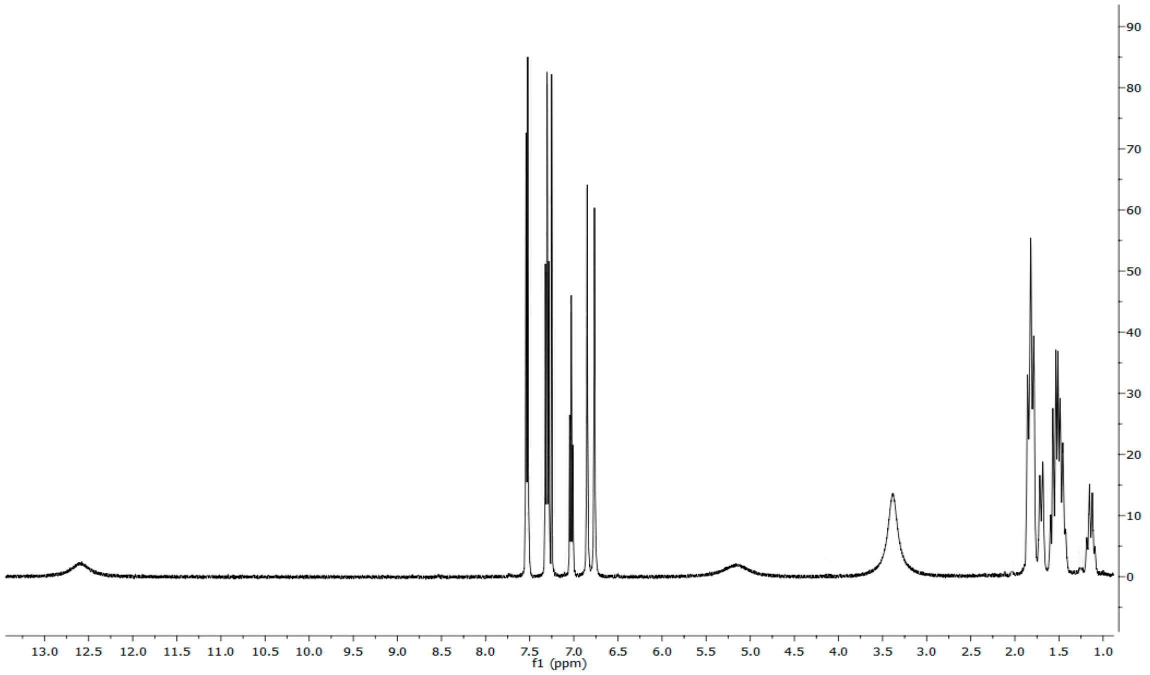

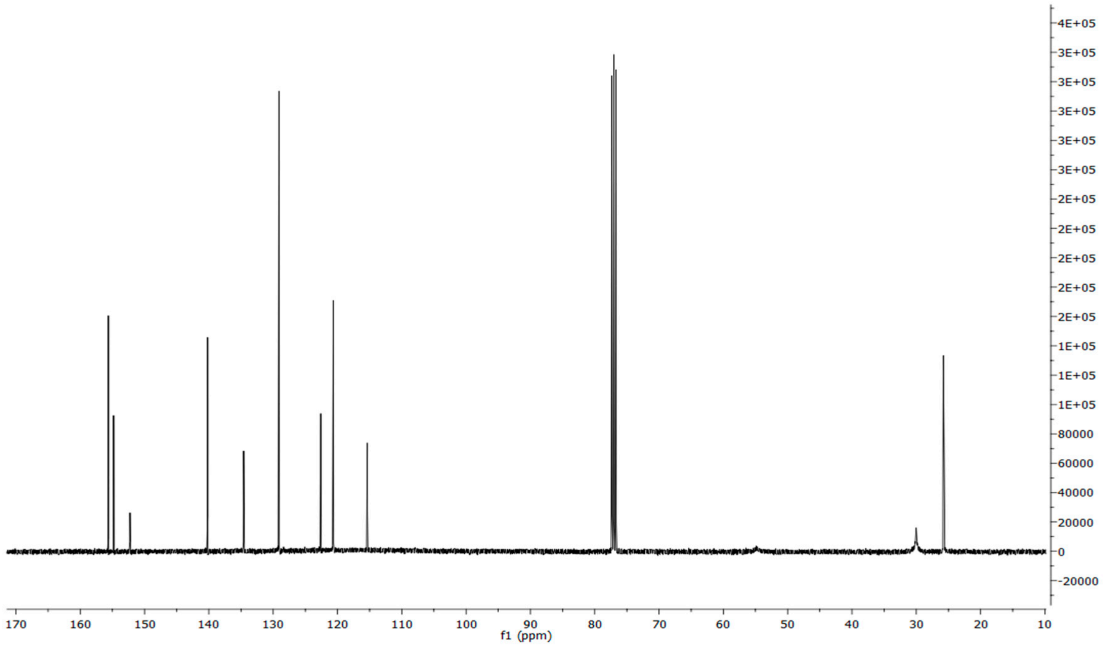

Fig. S3. ^{1}H- and ^{13}CNMR spectra ($CDCl_3$) of compound **3**.

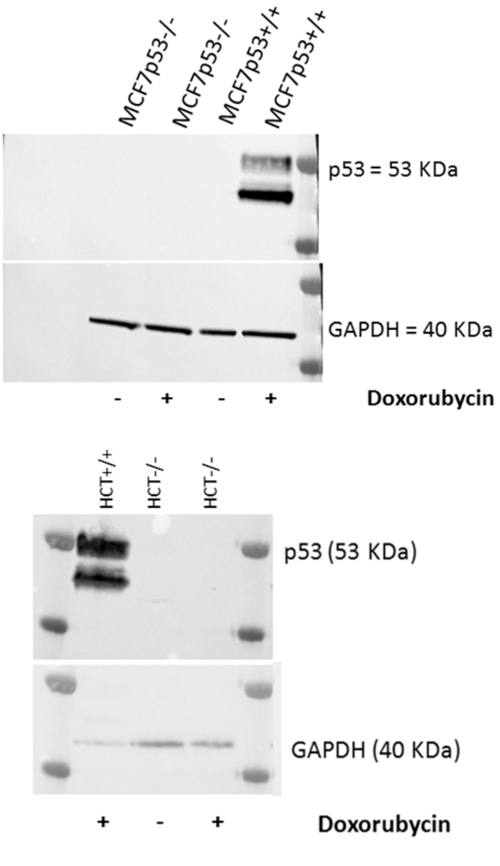

Fig. S4. Western Blot to control the presence or absence of p53 in MCF7 and HCT cell lines. Cells were treated with and without Doxorubicin, an activator of p53.

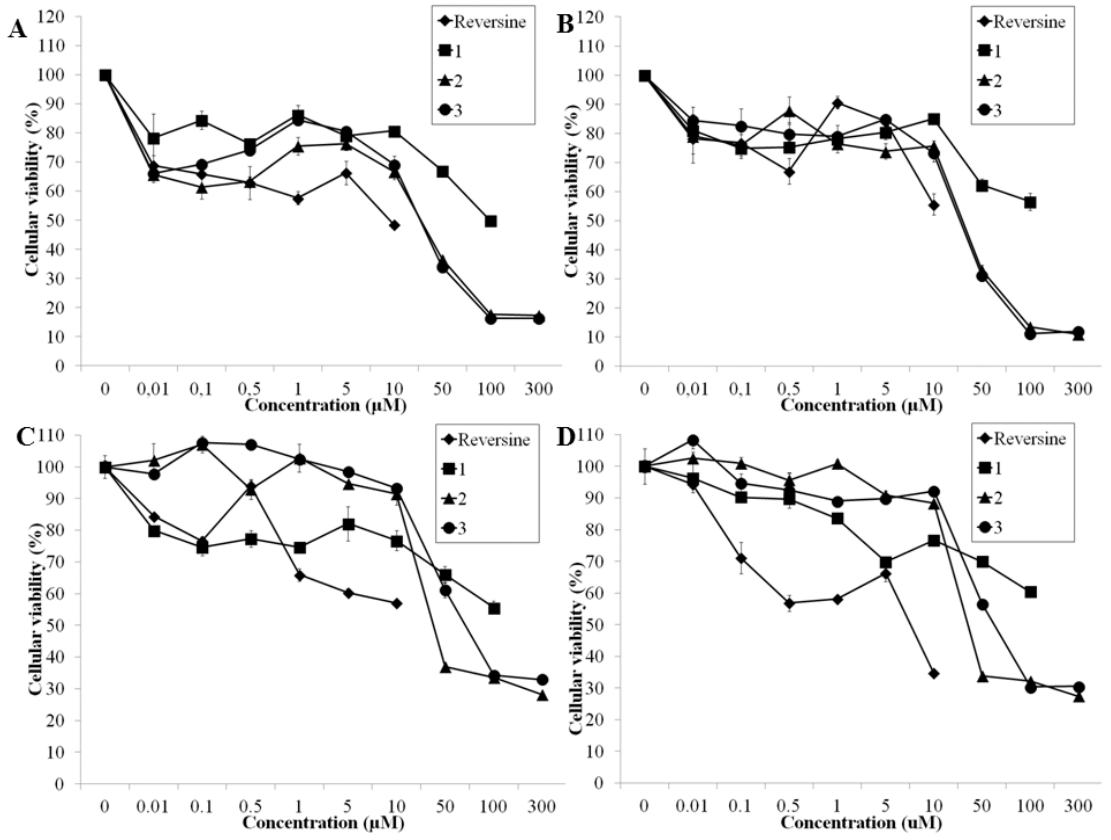

Fig. S5. Reversine and the related molecules **1-3** suppressed the growth of human cancer cells. A MCF-7 Vector, B MCF-7shp53, C HCT116p53$^{+/+}$ and D HCT116p53$^{-/-}$, were incubated with reversine or its analogues with multiple dosage for 24 hrs. Cell viability was determined by MTT assay. Results are the merge of three experiments.

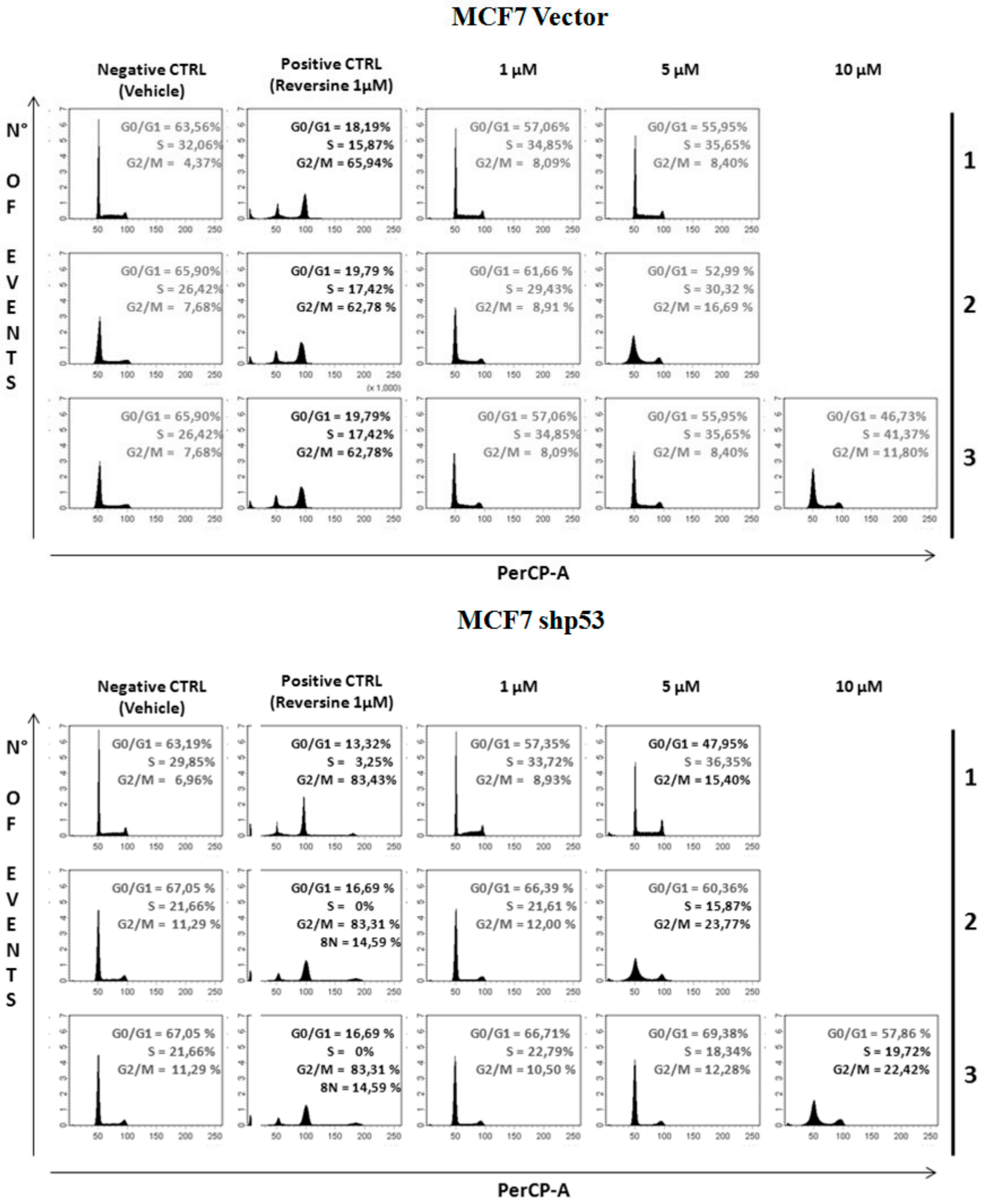

Figure S6. Representative images from analysis of MCF-7 Vector and MCF-7shp53 cell cycle after the treatment with different doses of compounds **1-3.** After the treatment a propidium iodide staining was performed and analyzed by flow cytometry.

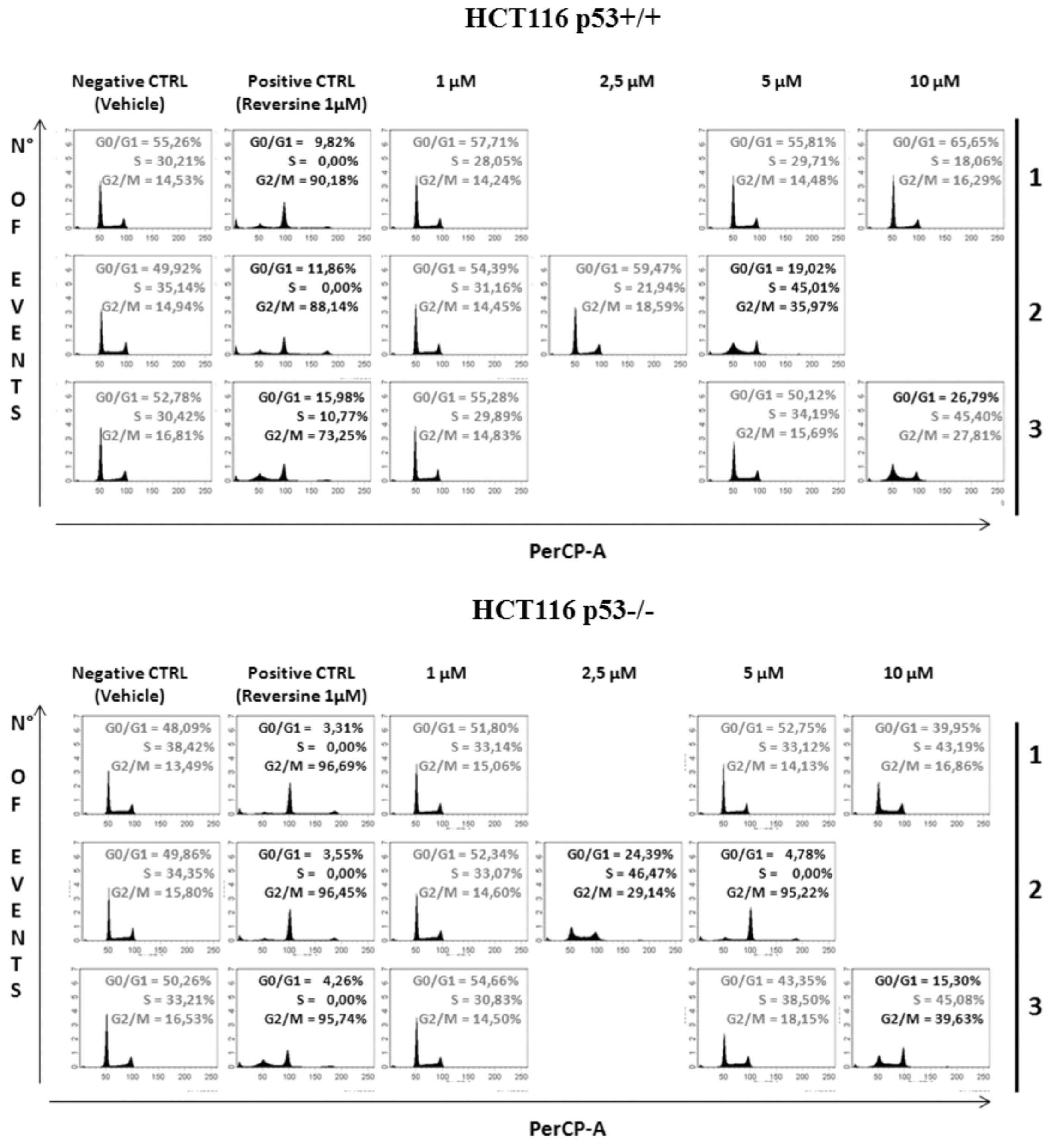

Figure S7. Representative images from analysis of HCT116 wt and HCT116 p53$^{-/-}$ cell cycle after the treatment with different doses of compounds **1-3**. After the treatment a propidium iodide staining was performed and analyzed by flow cytometry.

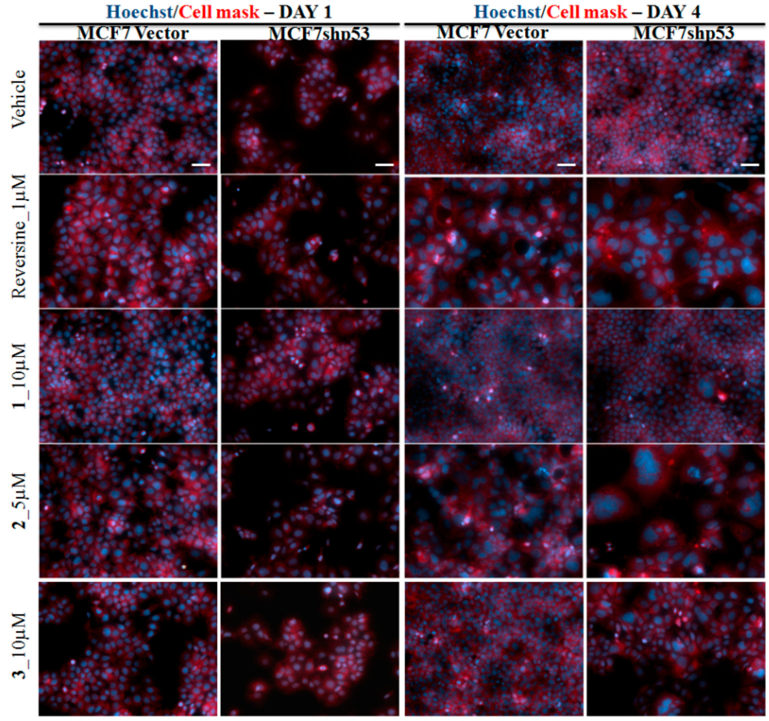

Figure S8. Nuclei and cell membrane staining on MCF-7 Vector and MCF-7shp53 after **1-3** treatments at day 1 and day 4 (Scale bar = 50µm).

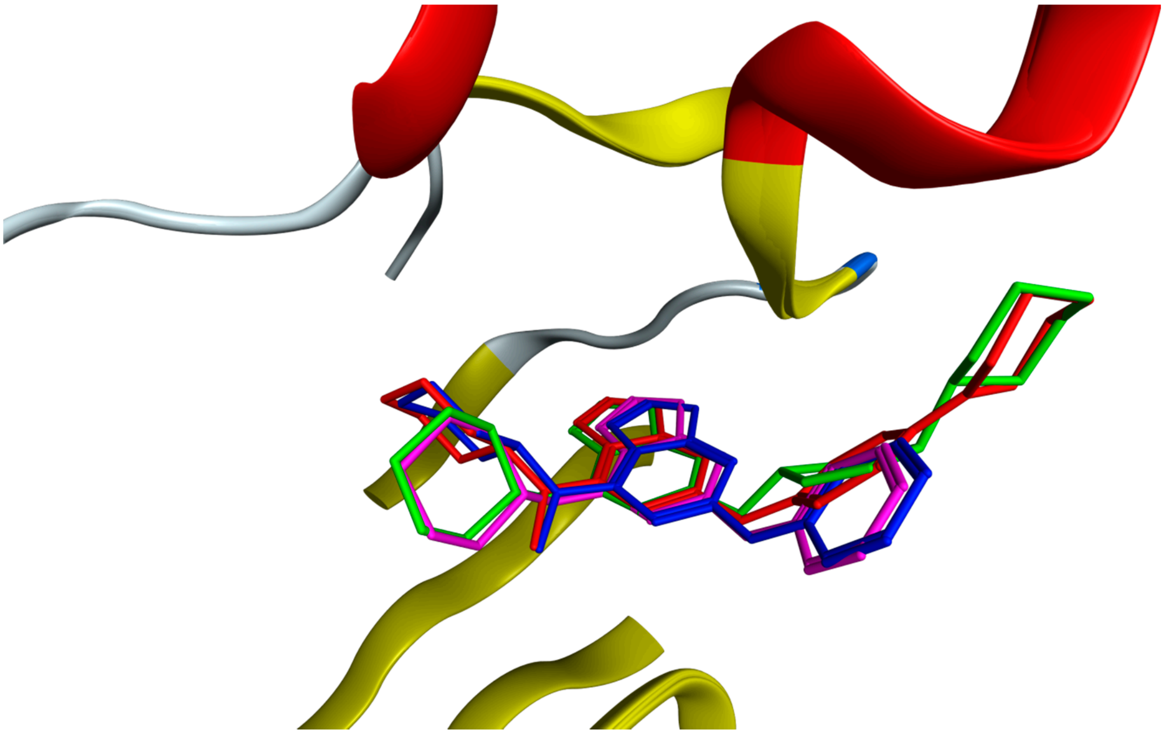

Figure S9. Superimposed structures of H*N*(9) tautomers of reversine (green) and molecules **1**(red), **2** (magenta) and **3** (blue) in the complexes with Aurora-B kinase (2VGO), as obtained by docking calculation.

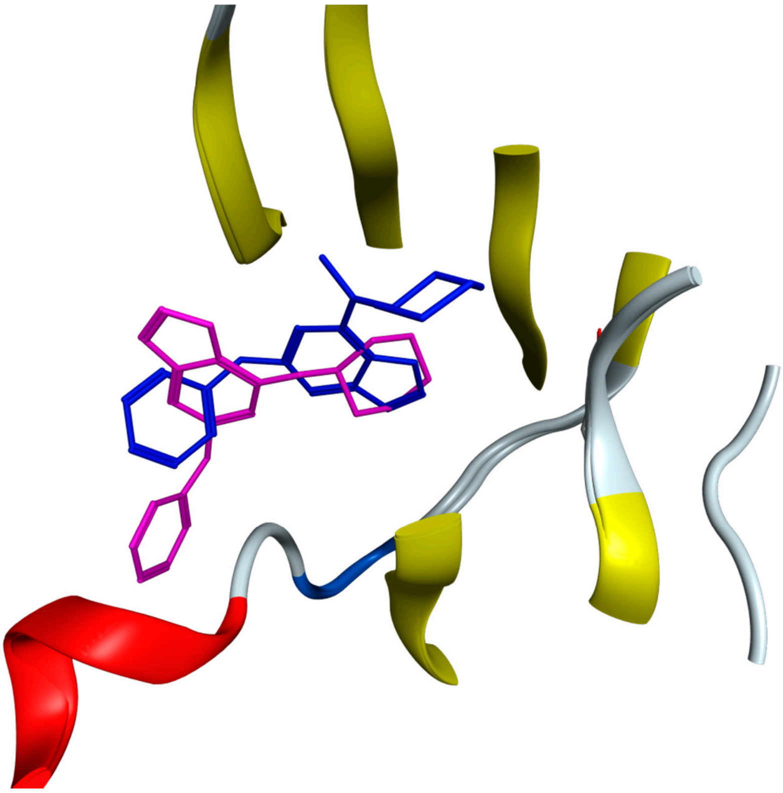

Figure S10. Superimposed structures of H*N*(7) tautomers of molecules **2** (magenta) and **3** (blue) in the complexes with Aurora-B kinase (2VGO), as obtained by docking calculation.
